# Single-cell correlations of mRNA and protein content in a human monocytic cell line after LPS stimulation

**DOI:** 10.1371/journal.pone.0215602

**Published:** 2019-04-19

**Authors:** Daniel M. Kalb, Samantha H. Adikari, Elizabeth Hong-Geller, James H. Werner

**Affiliations:** 1 Center for Integrated Nanotechnologies, Los Alamos National Laboratory, Los Alamos, New Mexico, United States of America; 2 Bioscience Division, Los Alamos National Laboratory, Los Alamos, New Mexico, United States of America; University of California Irvine, UNITED STATES

## Abstract

The heterogeneity of mRNA and protein expression at the single-cell level can reveal fundamental information about cellular response to external stimuli, including the sensitivity, timing, and regulatory interactions of genes. Here we describe a fully automated system to digitally count the intron, mRNA, and protein content of up to five genes of interest simultaneously in single-cells. Full system automation of 3D microscope scans and custom image analysis routines allows hundreds of individual cells to be automatically segmented and the mRNA-protein content to be digitally counted. Single-molecule intron and mRNA content is measured by single-molecule fluorescence in-situ hybridization (smFISH), while protein content is quantified though the use of antibody probes. To mimic immune response to bacterial infection, human monocytic leukemia cells (THP-1) were stimulated with lipopolysaccharide (LPS), and the expression of two inflammatory genes, IL1β (interleukin 1β) and TNF-α (tumor necrosis factor α), were simultaneously quantified by monitoring the intron, mRNA, and protein levels over time. The simultaneous labeling of cellular content allowed for a series of correlations at the single-cell level to be explored, both in the progressive maturation of a single gene (intron-mRNA-protein) and comparative analysis between the two immune response genes. In the absence of LPS stimulation, mRNA expression of IL1β and TNF-α were uncorrelated. Following LPS stimulation, mRNA expression of the two genes became more correlated, consistent with a model in which IL1β and TNF-α upregulation occurs in parallel through independent mechanistic pathways. This smFISH methodology can be applied to different complex biological systems to provide valuable insight into highly dynamic gene mechanisms that determine cell plasticity and heterogeneity of cellular response.

## Introduction

Gene and protein expression in response to external stimuli have been most commonly observed at the ‘bulk’ level, resulting in average values of expression over large numbers of individual cells. While these studies can be informative, understanding the heterogeneity of gene expression at the single-cell level provides increased contextual information about the kinetics and fundamental regulatory mechanisms of these genes.[1] Additionally, single-cell measurements are critical for applications where rare cells, or heterogeneity of gene expression within a population of cells, can lead to drastically different biological outcomes such as cellular response to cancer treatment and host immunity to pathogen infection.[2, 3]

A number of recent advances have enabled quantitative measurements of gene expression at the single-cell level. For most of these techniques, there is an intrinsic balance between the number of genes analyzed and the number of cells analyzed. Techniques that examine a relatively large number of genes, such as qPCR and single-cell sequencing methods, have demonstrated a high range of gene measurements (96 for qPCR; >20,000 for sequencing) with a limited number of single-cells examined (few-hundreds for qPCR; tens for sequencing).[4–7] Furthermore, these techniques are generally dependent upon amplification systems to increase the amount of signal for detection. There is no such thing as a noise-free amplifier and low counts of copies/cell can be particularly challenging to measure.[8, 9] This difficulty of accurately measuring low copy numbers is compounded by the fact that the vast majority of genes exhibit ‘bursting’ behavior, where a large fraction of cells exhibit low expression. Although correcting for the noise and bias of amplification systems at low copies is an active field,[9, 10] sensitivity is ultimately limited by the amplifiers themselves.

Imaging-based techniques such as single-molecule fluorescence in-situ hybridization (smFISH) are able to image thousands of cells with single-molecule sensitivity without amplification and provide spatial information, but are generally limited to examining a few (~2–4) genes at a time with probes whose fluorescence emission does not overlap. In smFISH, the target sequence of interest is targeted with many (~20–50) short oligonucleotide probes labeled with a single fluorophore.[11, 12] Following hybridization, a relatively the bright diffraction limited spot comprised from ~20–50 fluorophores can be imaged on a conventional fluorescence microscope. Complementary fluorescent quenchers have also been demonstrated to reduce the background of unbound fluorescent probes where signal to noise is key, such as the labeling of small RNA (sRNA) of limited nucleotide lengths (~200).[13] It is also worth noting the development of barcode labeling techniques that apply successive rounds of imaging and hybridization to increase the number of genes analyzed.[14–16]

Single-cell mRNA labeling techniques have revealed that the expression of many genes is a stochastic process, often characterized by large distributions of content,[7, 17, 18] which have previously been characterized as ‘gene expression noise.’ However, recent work has shown that unlike the traditional conception of ‘noise,’ these distributions of heterogeneous expression are informative and can yield fundamental information about the sensitivity, timing, and regulatory mechanisms of genes.[1, 19] Beyond the distribution shapes, correlations of mRNA and protein expression at the single-cell level provide additional insight into the regulatory mechanism (e.g. Does A make B? Does A repress B? Are A and B unrelated or controlled by another external factor?).

Here we describe an automated platform for the acquisition and quantification of gene content at the single-cell level using smFISH and apply this system to examine the expression of two inflammatory biomarkers, IL1β and TNF-α, in THP-1 cells following stimulation with lipopolysaccharide (LPS), a cell wall component of Gram negative bacteria. Multiple studies have already examined this relationship at the bulk level.[20, 21] IL1β and TNF-α are key mediators of host immunity during pathogen infection and other inflammatory conditions.[22] Prescription drugs targeting both IL1β and TNF-α have been demonstrated as effective tools for the remediation of pain and symptoms in a wide range of chronic inflammatory diseases such as rheumatoid arthritis[22–25], gout[22], diabetes[22, 26, 27], and Crohn’s disease[28]. In our single-cell studies, we found that mRNA expression of IL1β and TNF-α became correlated in response to LPS stimulation and that IL1β and TNF-α upregulation occurred through independent mechanistic pathways. Furthermore, one can incorporate the single cell data into quantitative predictions about the kinetics of gene regulation, including transcription initiation, transcription elongation, mRNA export, and mRNA decay.[29] Understanding the heterogeneity of IL1β and TNF-α responses at the single-cell level will provide further insights into the regulatory mechanisms of these genes and potentially lead to the development of more effective treatments for infectious and inflammatory diseases.

## Materials and methods

### Acquisition hardware

A conventional wide-field microscope (Olympus IX71), arc lamp (Olympus U-RFL-T), and high NA objective (Olympus UAPON: 1.49 numerical aperture 100X oil immersion) are used to excite and image the fluorescent mRNA, intron, and protein data in three dimensions, with scanning performed in XY with a 2D Thorlabs stage (BSC102) and Z-scanning performed with a Z Piezo (Physik Instrumente, PI-721.20). Images are acquired with a Hamamatsu ORCA-Flash 4.0 camera with appropriate exposure times, as short as 10 ms for DAPI and as long as ~1000 ms for the mRNA-FISH stains, depending upon signal levels. All acquisition hardware is automated in LabVIEW to acquire Z-scans across a wide XY space, resulting in images of thousands of individual cells per hour. Optical filters are chosen to best match the excitation/emission spectra of the fluorescence probes including DAPI (Semrock 1160B), FITC (Semrock 3540B), Quasar 570 (Chroma 49304), CAL Flour Red 610 (Chroma 49306), Quasar 670 (Chroma 49009), and a bright field channel. Up to five fluorescent channels as well as a bright field image are acquired at each spatial point and the raw image data is stored for offline analysis.

### Software: mRNA counting and image segmentation

A custom MATLAB script is used to count the number of mRNA in each cell. In summary, this routine: 1) automatically finds and segments the individual cells based upon a bright-field image, the nuclear stain (DAPI), and the smFISH channel; 2) filters the image to help detect near-diffraction limited spots using a Laplacian of a Gaussian filter; 3) thresholds the filtered image data (S1 Fig); 4) fits all of the mRNA ‘spots’ using a GPU accelerated algorithm, and 5) assigns all ‘spots’ to the segmented cells and exports data for further analysis. This process is repeated to quantify the intron bursting sites, with different LOG filter parameters applied and further thresholding controls implemented to ensure accurate selection of bursting sites.

### Segmentation

**Fig 1:**
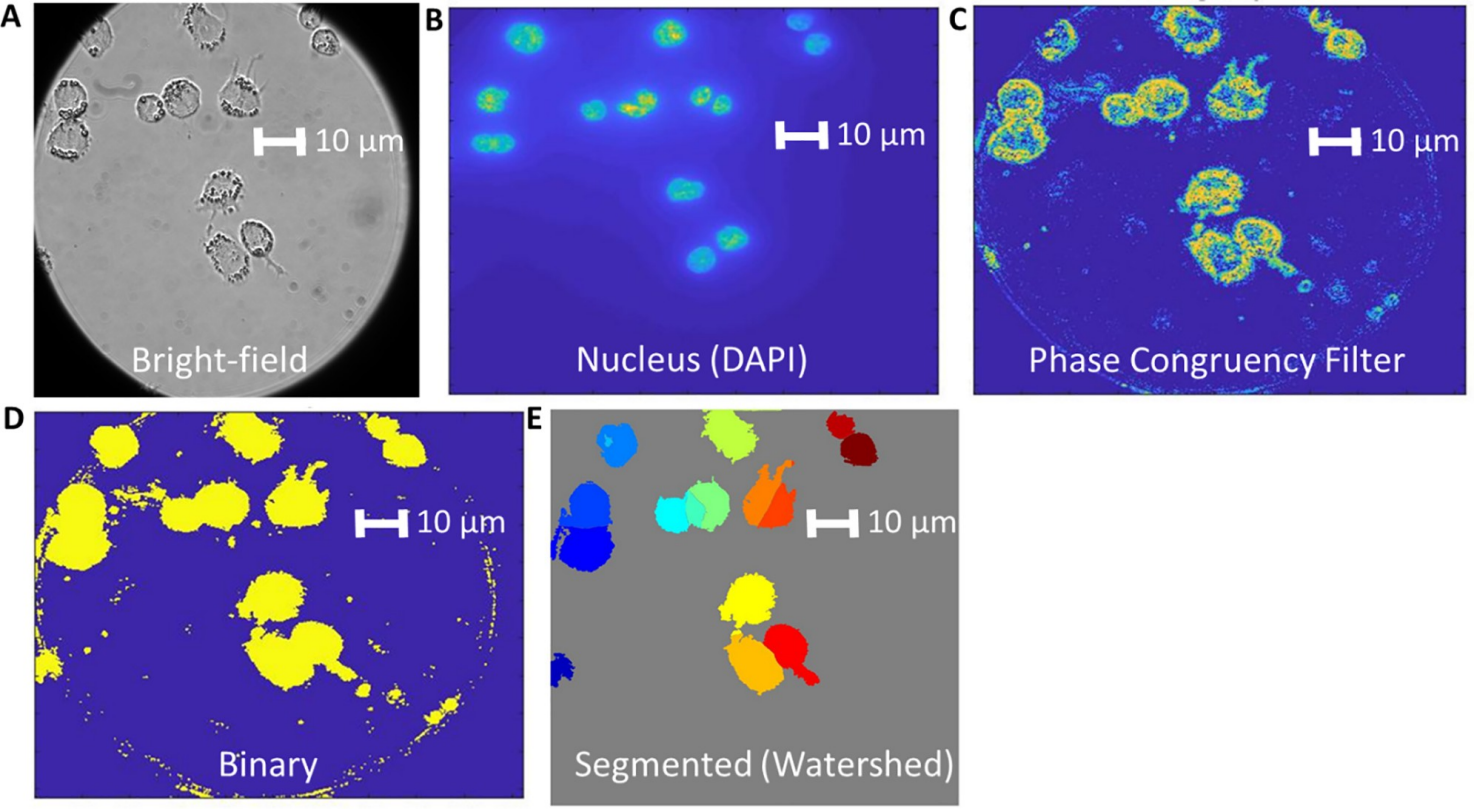
Automated cell segmentation process. The bright-field image (A) and nucleus data from a DAPI stain (B) are utilized to segment the cells. The combination of the DAPI data, the smFISH channel, and the phase congruency filtered bright field data (C) are utilized to create a binary image (D). A watershed from the nucleus data and the binary image are utilized to create the final segmented image (E).

As shown in Fig 1, our algorithm utilizes three images at each XY position (DAPI, Bright Field, and smFISH channel) to segment and outline each individual cell. Firstly, a conventional edge detection and watershed filter is used on the DAPI image to find and count each individual nuclei. Generally, this channel shows high signal/noise and clear edges of the nuclei, as cell plating is done at a density enabling spatially distinct (not overlapped) nuclei. The segmentation of the nuclei serves two purposes: 1) it enables counting mRNA and intron contents inside verses outside nuclei and 2) it labels and counts each spatially distinct nuclei as an individual cell to begin the segmentation process. Following identification and counting of each individual nuclei, a phase congruency filter is utilized to outline the edges of the cell from the bright-field image data. Additionally, the fluorescent channel from the smFISH channel is filtered (Laplacian-of-Gaussian) and thresholded to ensure that spatial locations with high gene expression and fluorescent staining are included within the cell boundaries.Next each of the three channels (DAPI, Bright-field, and smFISH) are individually thresholded to create three binary images. All three binary images are added together, yielding a single image with intensity values between 0–3. Anything greater than or equal to 1 is considered part of a cell and is again thresholded to form another binary image. Some basic image transforms are performed (dilations, erosions, median filter, and void filling), and the cells are watershed from their central nuclei (DAPI channel) to their edges to create the final masks. Comparison of this automated process to a manual segmentation are consistent with each other, both in terms of total number of cells counted and cellular mRNA counts, to within a few percent (S2–S4 Figs, S1 Table).

### Cell culture

A human monocytic cell line THP-1 (ATCC, TIB-202) was cultured in a humidified incubator with 5% CO_2_ at 37°C in R10% medium: *RPMI-1640 Medium* (with glutamine, no phenol red, Gibco) supplemented with 10% fetal bovine serum (FBS, ATCC). Cells were passaged every 5 days, and used for experiments from age 90–180 days.

### RNA extraction

10^6^ THP-1 cells were seeded into 6cm sterile dishes with 100nM phorbol 12-myristate 13-acetate (PMA, Sigma) for 48hrs at 37°C. Cells were stimulated with 0.5mg/mL lipopolysaccharide (LPS, *E*. *coli* O55:B5, Sigma) and incubated for the indicated time-points up to 8hrs at 37°C. Cells were washed in PBS (Invitrogen) and lysed in 500μL *RLT Buffer* (Qiagen) containing 1% beta-mercaptoethanol (Sigma). Total RNA from 10^6^ THP-1 cells was extracted using the *RNeasy Mini Kit* (Qiagen), eluted in 50μL RNase-free distilled water (Invitrogen), and treated with DNase for 1hr at 37°C using the *Turbo DNA-free kit* (Invitrogen). RNA concentration was measured using a NanoDrop spectrometer, and the RNA was then stored at -80°C.

### Quantitative PCR

Quantitative PCR (qPCR) was performed in 96-well optical reaction plates (Applied Biosystems) using *Taqman RNA-to-CT One-Step Kit* (Applied Biosystems) with the following *Taqman* qPCR probes (with FAM-MGB reporter): human TLR4 (# Hs00152939_m1, Amplicon length: 89), human IL1-beta (# Hs01555410_m1, Amplicon length: 91), human TNF-alpha (# Hs00174128_m1, Amplicon length: 80), human GAPDH (# Hs99999905_m1, Amplicon length: 122), and human Actin-beta (# Hs99999903_m1, Amplicon length: 171). Each reaction contained 40ng RNA in 25μL final volume. Reverse transcription was run for 30min at 48°C.

### Slide preparation and RNA smFISH staining

Chambered cover-glass slides (#1.0 borosilicate glass, 8 wells, Lab-Tek) were coated with a sterile bovine fibronectin solution (1 μg/well in PBS, Sigma) overnight at 4°C. 10^5^ THP-1 cells/well were seeded onto slides in R10% medium containing 100nM PMA for 48hrs at 37°C. Cells were serum-starved in *RPMI-1640 Medium* (no FBS) for 2hrs at 37°C and stimulated with 100μg/well LPS for the indicated time-points at 37°C. Cells were washed in PBS, fixed in paraformaldehyde (4% solution in PBS (v/v), Alfa Aesar) for 15min, washed twice in PBS, and permeabilized in 70% ethanol in RNase-free distilled water (v/v) (ThermoFisher) for at least 1hr at 4°C, up to 24hrs. Cells were then washed in PBS, blocked in bovine serum albumin (2% solution in PBS (v/v), ThermoFisher) for 1hr, washed in PBS and then in *RNA FISH Wash Buffer A* (Stellaris) for 20 min. Unstimulated cells were washed and fixed at t = 0hrs after serum starvation.

Cells were stained with the indicated combinations of custom-designed RNA FISH probes (Stellaris, details in supplemental information S1 File) and/or antibodies (multiple sources, details below). Probes and antibodies were diluted in *RNA FISH Hybridization Buffer* (Stellaris/Biosearch Technologies) to 100nM for mRNA probes and intron probes, 2μg/mL for primary antibodies, and 1μg/mL for secondary antibodies, then incubated with 100μL/well for 4hrs at 37°C. Staining conditions were made in duplicate on each slide. Following probe hybridization, cells were washed three times in *RNA FISH Wash Buffer A* for 30min each time at 37°C, stained with 100ng/mL DAPI solution (ThermoFisher) in *RNA FISH Wash Buffer A* for 20min at 37°C, and washed in *RNA FISH Wash Buffer B* (Stellaris) for 20min. Cells were then washed in PBS and stored in 200μL/well *SlowFade Gold Anti-Fade Mountant* (ThermoFisher), diluted 4x in PBS, for up to 7 days at 4°C. Unless otherwise specified, all steps were performed at room temperature, incubations were performed with 250μL/well, and wash steps were performed with 500μL/well.

Antibodies used in analysis include: (1) Anti-TNF-alpha, mouse monoclonal antibody [clone 52B83] (Abcam); (2) Anti-IL1-beta, rabbit polyclonal antibody (Abcam); (3) Anti-IL1-beta (Pro-form), rat monoclonal antibody [clone NJTEN3], APC (ThermoFisher); (4) Anti-rabbit IgG H&L, goat antibody, Alexa Fluor 647 (Abcam); and (5) Anti-mouse IgG H&L, goat antibody, Alexa Fluor 488 (Abcam).

## Results

### Kinetic response of IL1β and TNF-α over time

**Fig 2:**
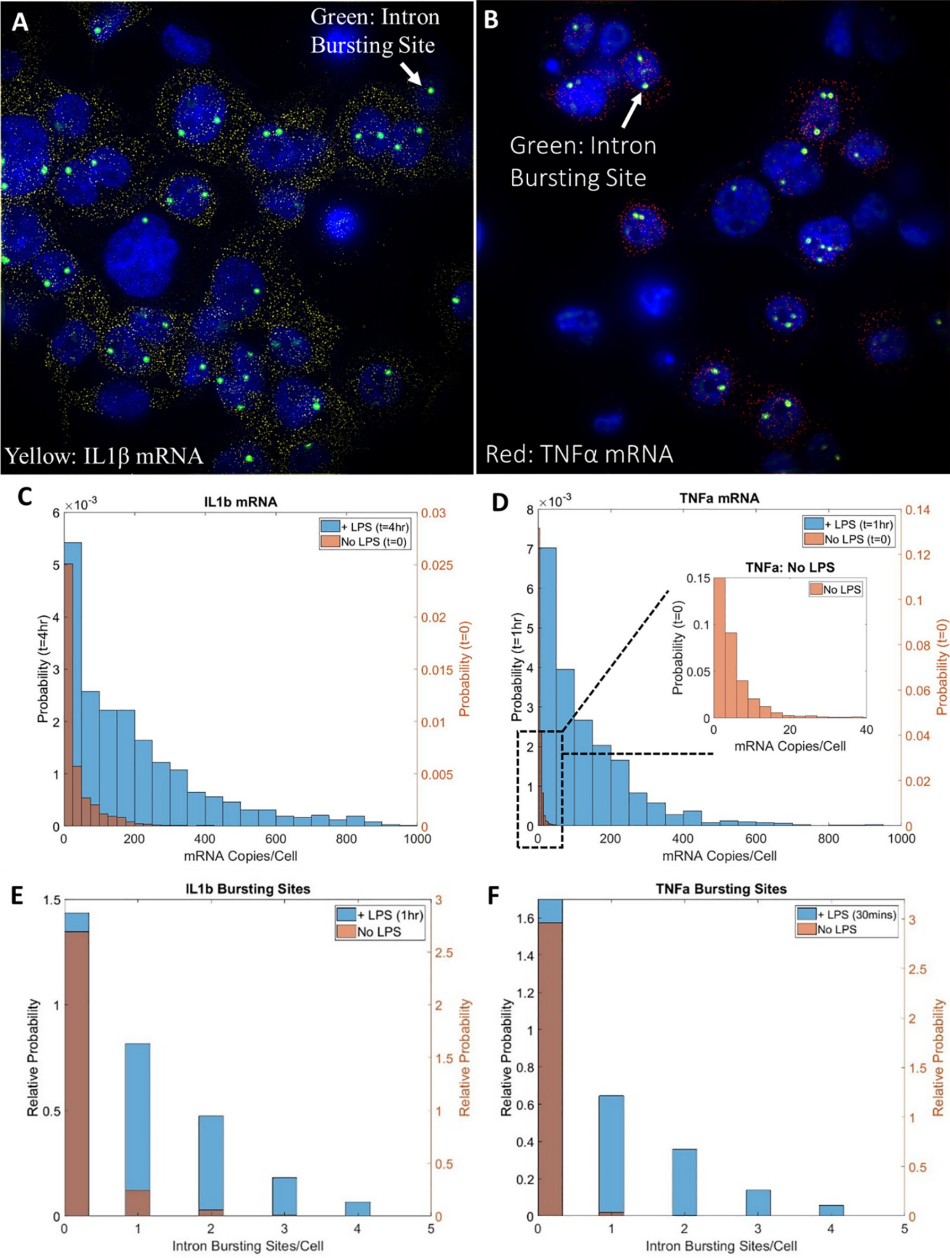
Single-cell gene expression of IL1β and TNF-α. Example images of mRNA (small yellow/red spots) and intron bursting site data (large green spots) for IL1β (A) and TNF-α (B) after 1hr of LPS stimulation overlaid with nuclear content (DAPI, blue). Each small yellow spot is a single IL1β mRNA, each red spot is a single TNF-α mRNA, while each large green spot is an intron bursting site where many (~100) copies of IL1β are being actively transcribed. Distributions of single-cell mRNA counts for IL1β (C) and TNF-α (D) are upregulated with the addition of LPS. Intron bursting site distributions are also upregulated following the addition of LPS for both IL1β (E) and TNF-α (F). Note that cells identified with 3 or 4 intron bursting sites result from errors in cell segmentation or come from cells that are undergoing mitosis.

**Fig 3:**
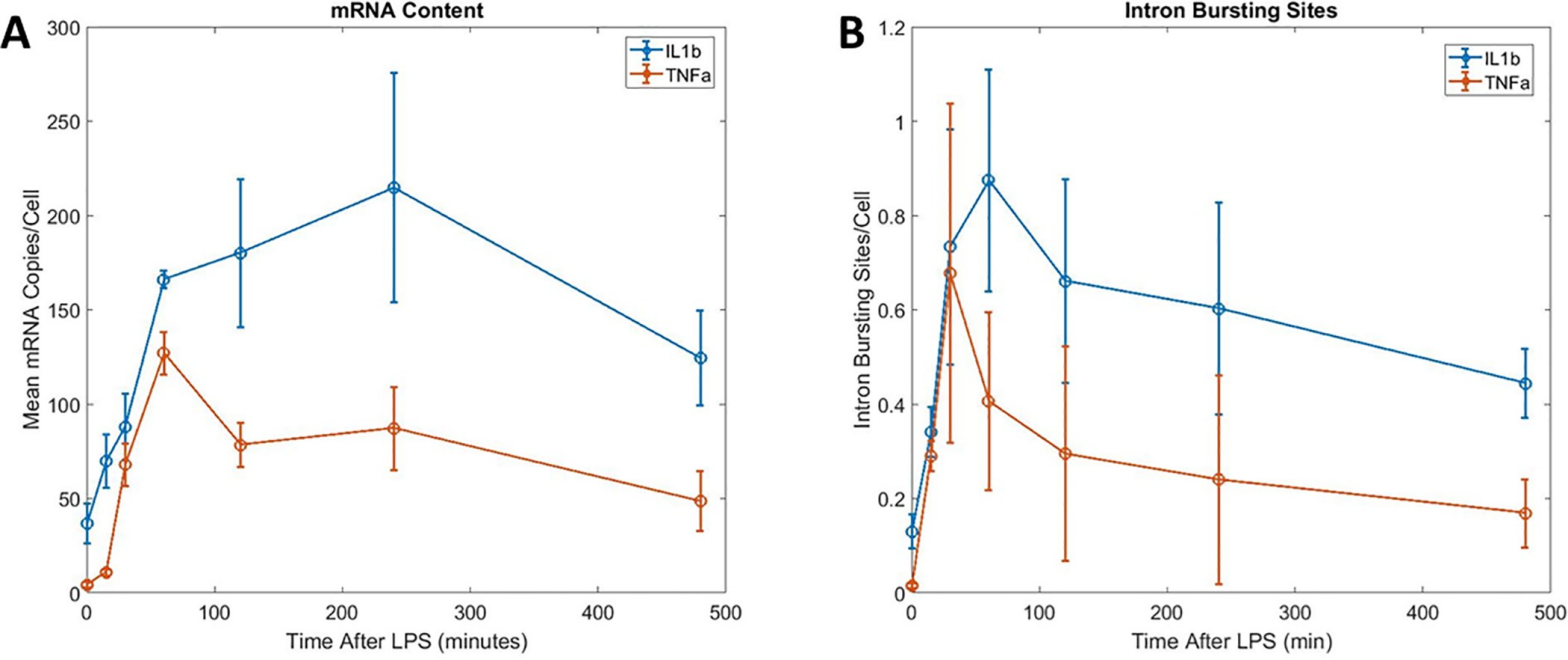
**Mean mRNA content (A) and intron bursting sites (B) over time for IL1β and TNF-α.** Error bars are from the standard deviation of the mean measured from three or four scans (of approximately 1000 cells) over 2 wells of biological replicates. (We note that the breadth of the single cell mRNA distributions are much larger than the standard deviation of the mean shown as error bars above).

We applied smFISH and immunofluorescence to image the intron, mRNA, and protein expression of IL1β and TNF-α in THP-1 cells in response to LPS stimulation over time. (Fig 2A and 2B) The single-cell distributions of gene expression are characterized by the both the shape (Fig 2C–2F) and means of expression (Fig 3). These distributions can be fit reasonably well by both log-normal and gamma functions, while a Poisson distribution fits the data poorly (S5 and S6 Figs). Despite shifts in both mean and shape, the single-cell distribution of mRNA content is indicative of ‘bursting behavior’ across all time-points.

In the first 60 min of LPS stimulation, transcription of both IL1β and TNF-α rapidly increased to a mean of ~130 and ~170 copies mRNA/cell, respectively.(Fig 3) After 60 min, IL1β mRNA levels continued to increase to a maximum of ~210 copies mRNA/cell at 240 min and then tapered off to 130 copies by 480 min. In contrast, TNF-α expression decreased after 60 min to a mean of 80 copies/cell at 120 min and plateaued at that level through 480 min. Similar trends are shown with additional biological replicates in the supporting information (S7 Fig).

We also observed intron bursting sites (Fig 2, large green spots) that represent many (~100) copies of a transcribed gene. Comparing the integrated fluorescence intensity of the intron bursting sites to single intron copies leads to estimates that each bursting sites contains ~100 copies of the gene (S8 Fig). In the first 30 min, we saw a rapid increase in the number of intron bursting sites for both IL1β and TNF-α (Fig 3). While IL1β continued to increase and peak at ~60 min with ~0.9 intron bursting sites/cell, the number of TNF-α bursting sites reached a maximum of ~0.7 sites/cell at ~30 minutes of exposure before beginning to fall off.

**Fig 4:**
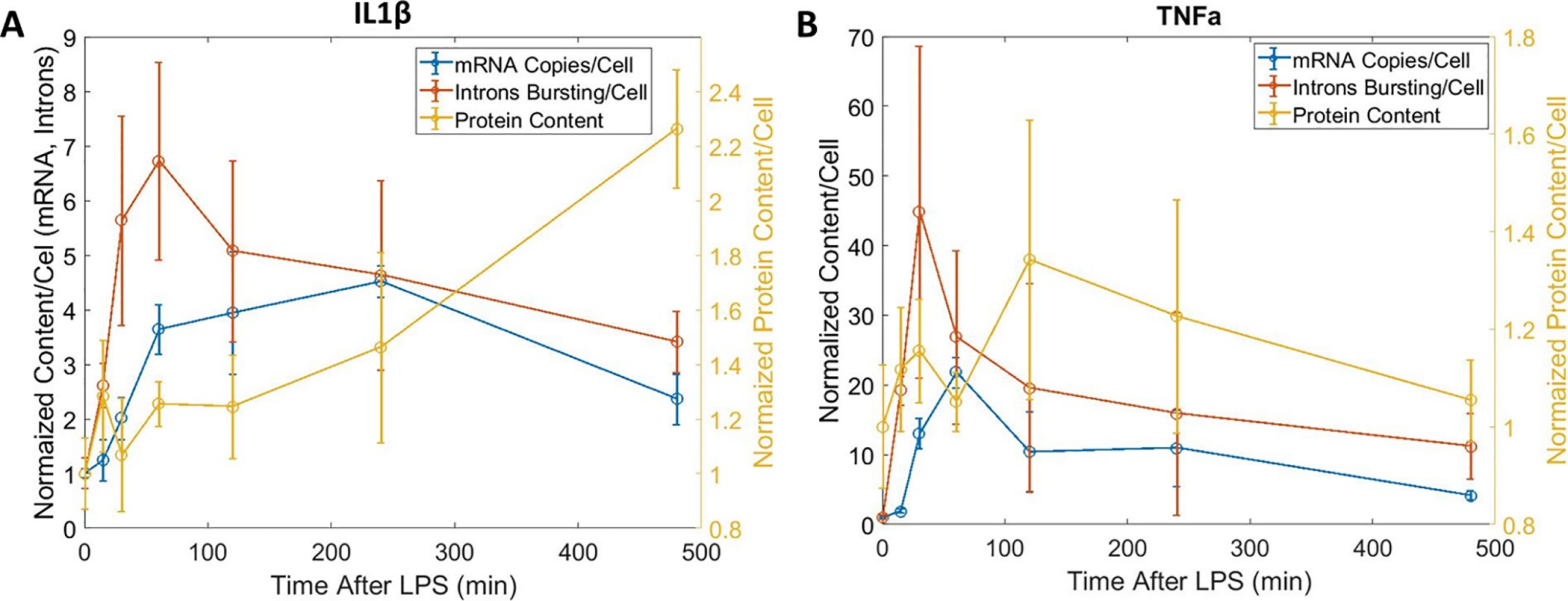
Mean mRNA, intron bursting site, and protein content for IL1β (A) and TNFα (B) over time.

The progressive maturation of each gene (intron-mRNA-protein) was examined by multicolor labeling and imaging both mRNA and protein content over time.(Fig 4) IL1β showed a progression of gene expression, with the upregulation of intron bursting sites, followed by mRNA, then finally protein. While TNF-α shows a significant progression from intron bursting sites to mRNA, only a modest increase in intracellular protein was observed.

### Correlations over time (single-cell: mRNA-mRNA, mRNA-protein)

**Fig 5:**
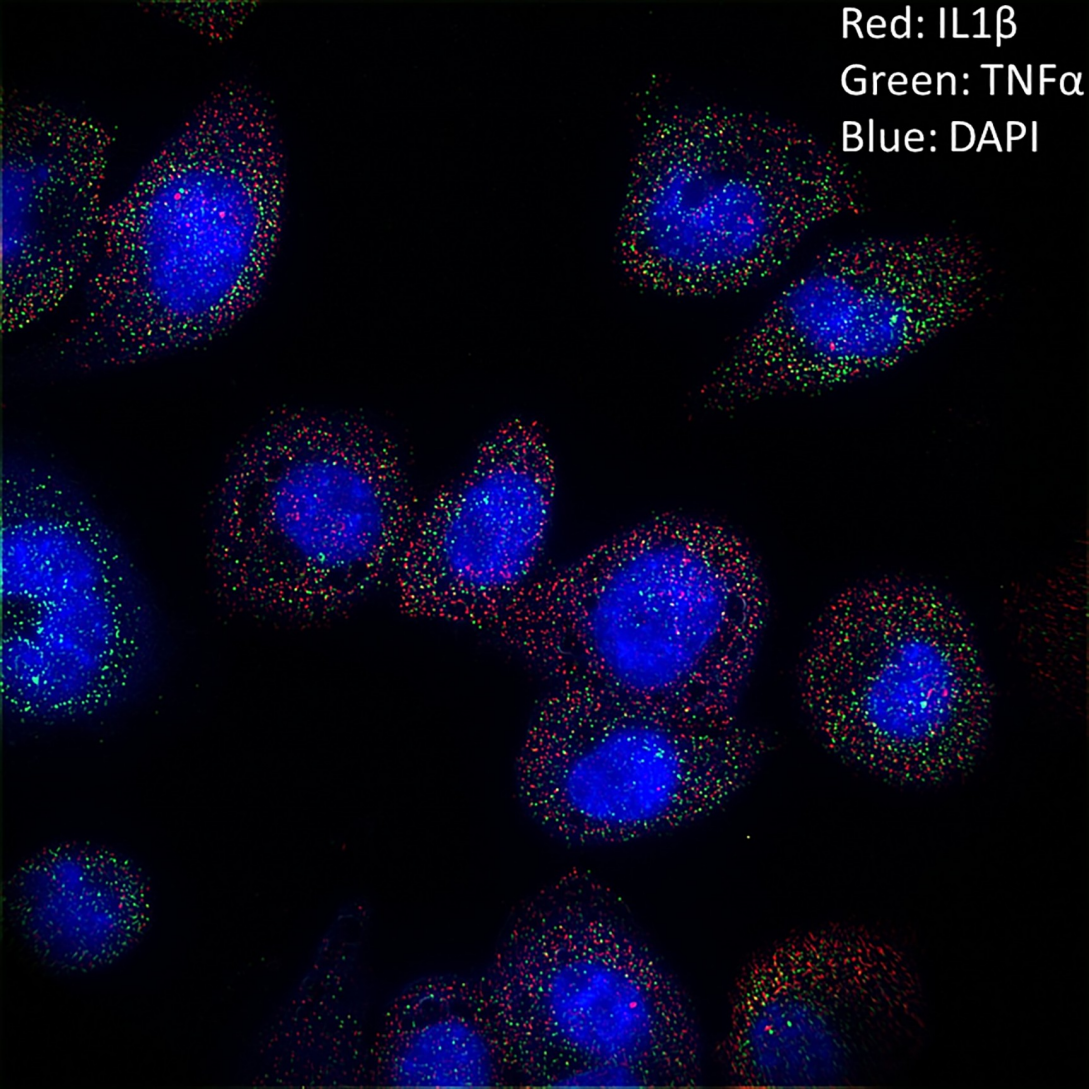
Simultaneous mRNA imaging of IL1β and TNF-α for single-cell corrolation of mRNA content. This image was taken after 1 hr of LPS stimulation and exhibited relative high expression of both TNF-α (green) and IL1β (red).

**Fig 6:**
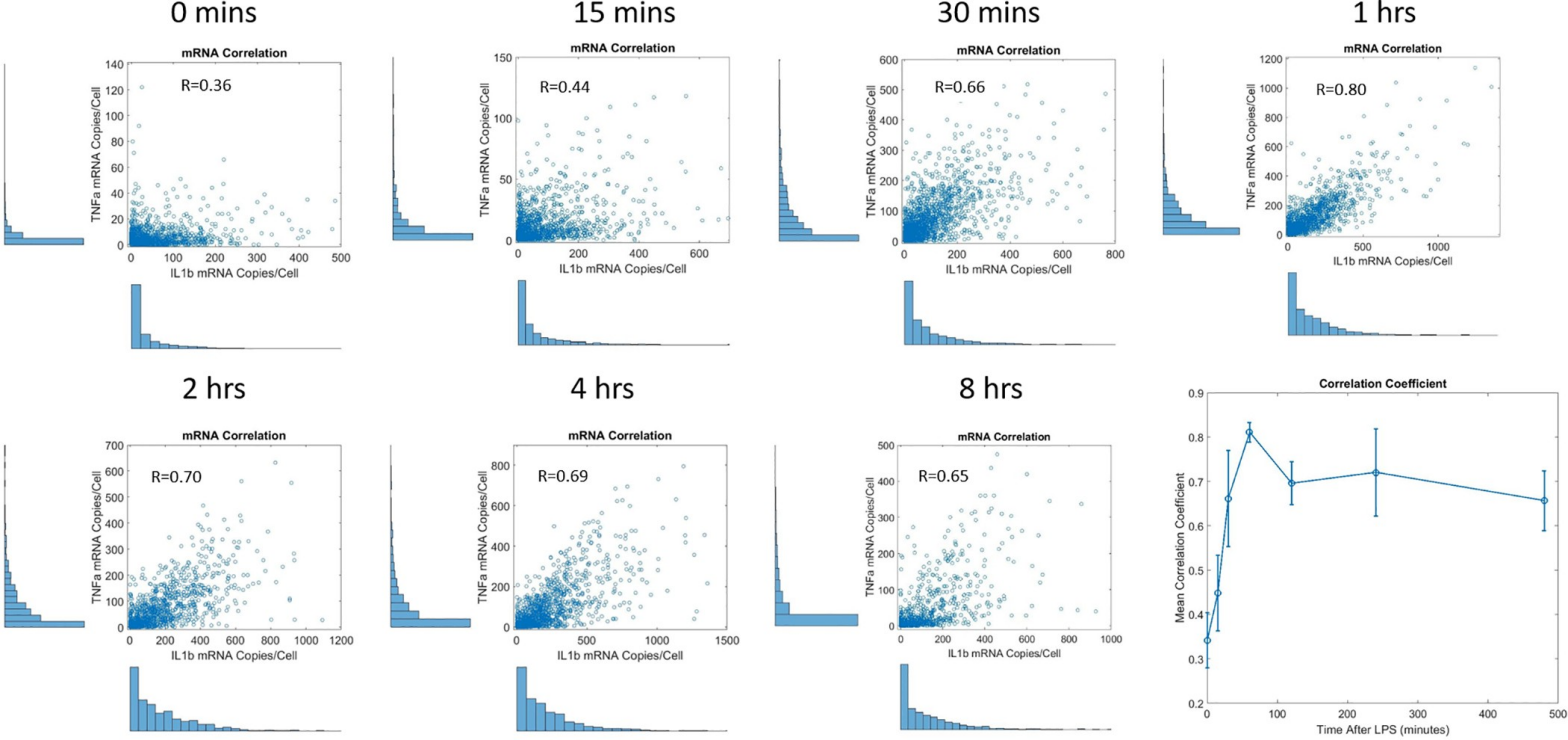
Scatter plots of IL1β-TNF-α mRNA correlation at the single-cell level. Each dot represents a single-cell). Without stimulation, the genes are weakly correlated. Correlation peaks at 1 hour when both of these inflammation genes are highly active.

Beyond kinetic information, simultaneous imaging of multiple genes and multiple molecules (including mRNA, introns, and protein) of interest allows for correlations of gene expression at the single-cell level to be explored. Simultaneous imaging of IL1β and TNF-α mRNA (Fig 5) was utilized to extract the correlation of mRNA expression over time (Fig 6).

As can be seen in Fig 6, initially, the correlation between IL1β and TNF-α mRNA is weak (correlation coefficient of 0.36). However, after LPS stimulation, both genes are turned on within a similar timescale and a positive correlation begins to emerge and become more prevalent. Between 4–8 hours, the genes are slowly becoming less correlated, which could be due to the fact that while both IL1β and TNF-α are activated at similar timescales, IL1β signaling continues for sensitized cells, with overall mRNA expression peaking later.[30] We emphasize that while there is a correlation amongst any pair of mRNAs due to the fact there is a correlation between cell size and mRNA expression (e.g. larger cells contain more copies of a given mRNA), this sized-based correlation is weaker than the correlation between mRNAs exhibited with cells at peak correlation (1hr LPS) (S9 Fig). Moreover, the fact the correlation between the two genes changes as a function of time following LPS exposure further indicates that these correlations are not simply due to cell-size related effects.

**Fig 7:**
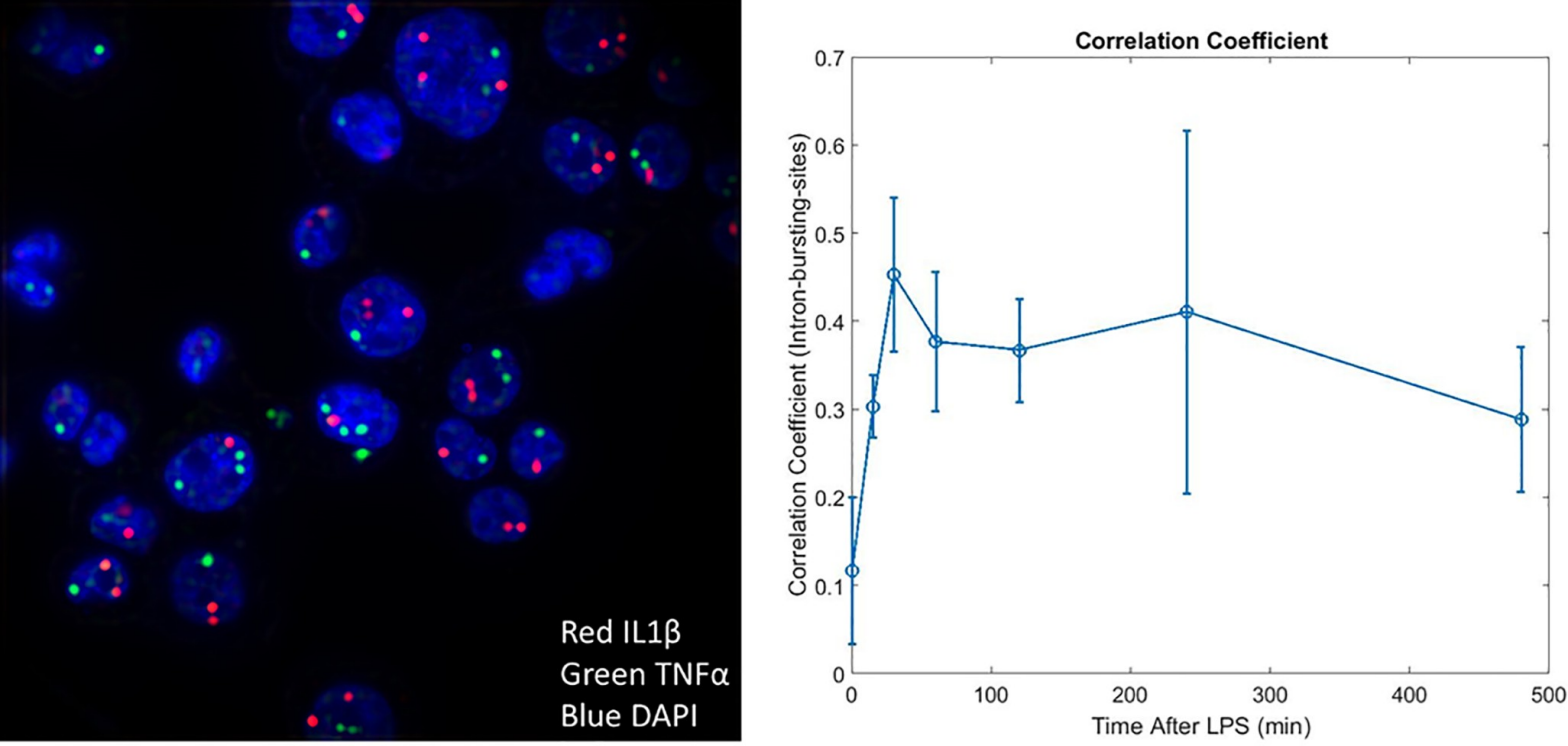
Correlation of intron bursting sites. The correlation peaks at 30 minutes when gene transcription is highly active. We note that this peak in correlation occurs earlier than the mRNA correlations. (Filtered intron image, raw data shown in S10 Fig).

In addition to mRNA-mRNA correlations, we also can explore the correlation between intron bursting sites for IL1β and TNF-α. We first note that the intron bursting sites of the two genes are spatially distinct from each other (see Fig 7), which was expected given that IL1β is encoded on chromosome 2 while TNF-α is encoded on chromosome 6. Much like the mRNA-mRNA correlations, the single-cell correlation of intron bursting sites for IL1β and TNF-α are initially uncorrelated (Fig 7), but display some correlation as expression of bursting sites peaks. However, since the correlation of these bursting sites never gets particularly large, we hypothesize that this correlation is in large part due to the similar timescale of the kinetic turn-on for both IL1β and TNF-α.

**Fig 8:**
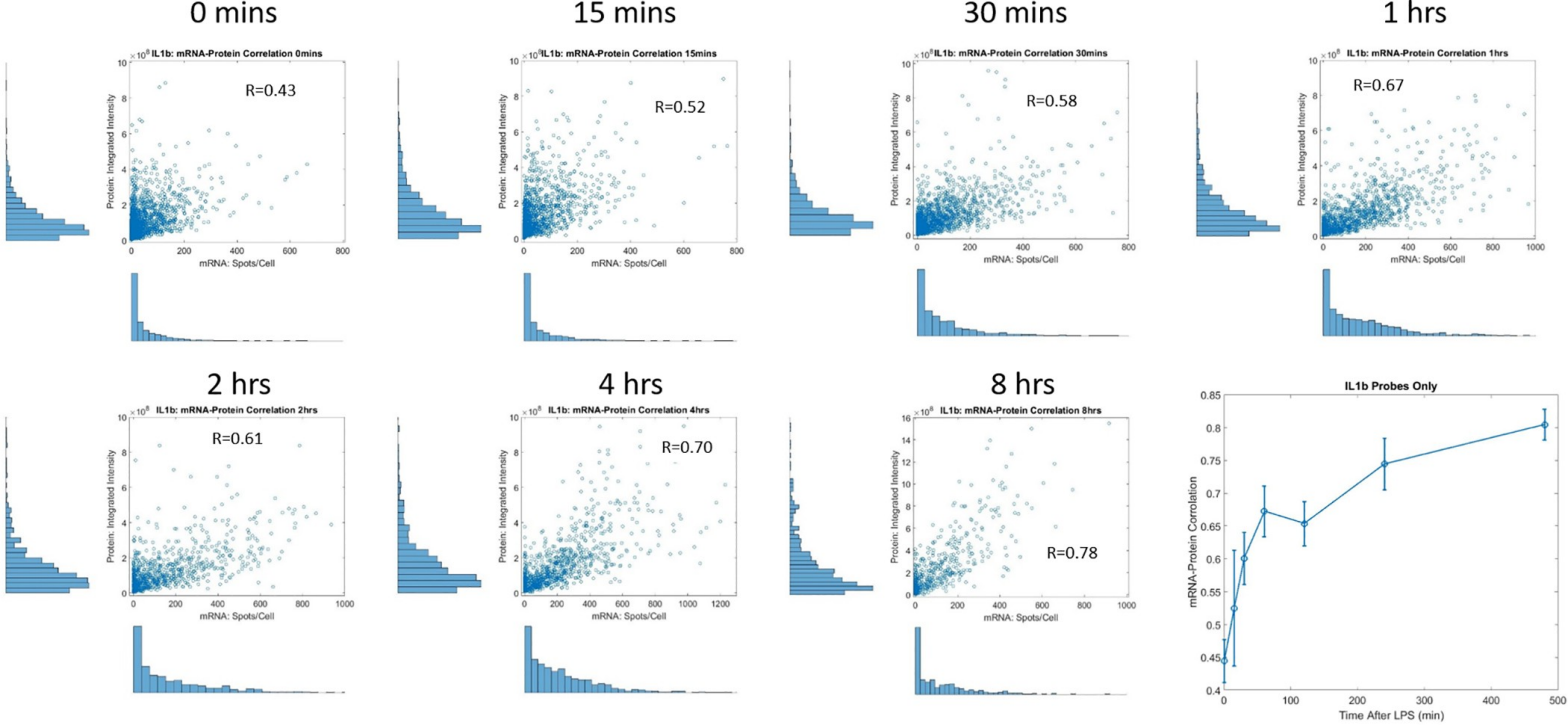
Scatter plot of mRNA-Protein correlation for IL1β and plot of correlation coefficient over time. Without LPS, there is a weak correlation between mRNA and protein content for IL1β. After LPS stimulation we see an increase in correlation between the two.

**Fig 9:**
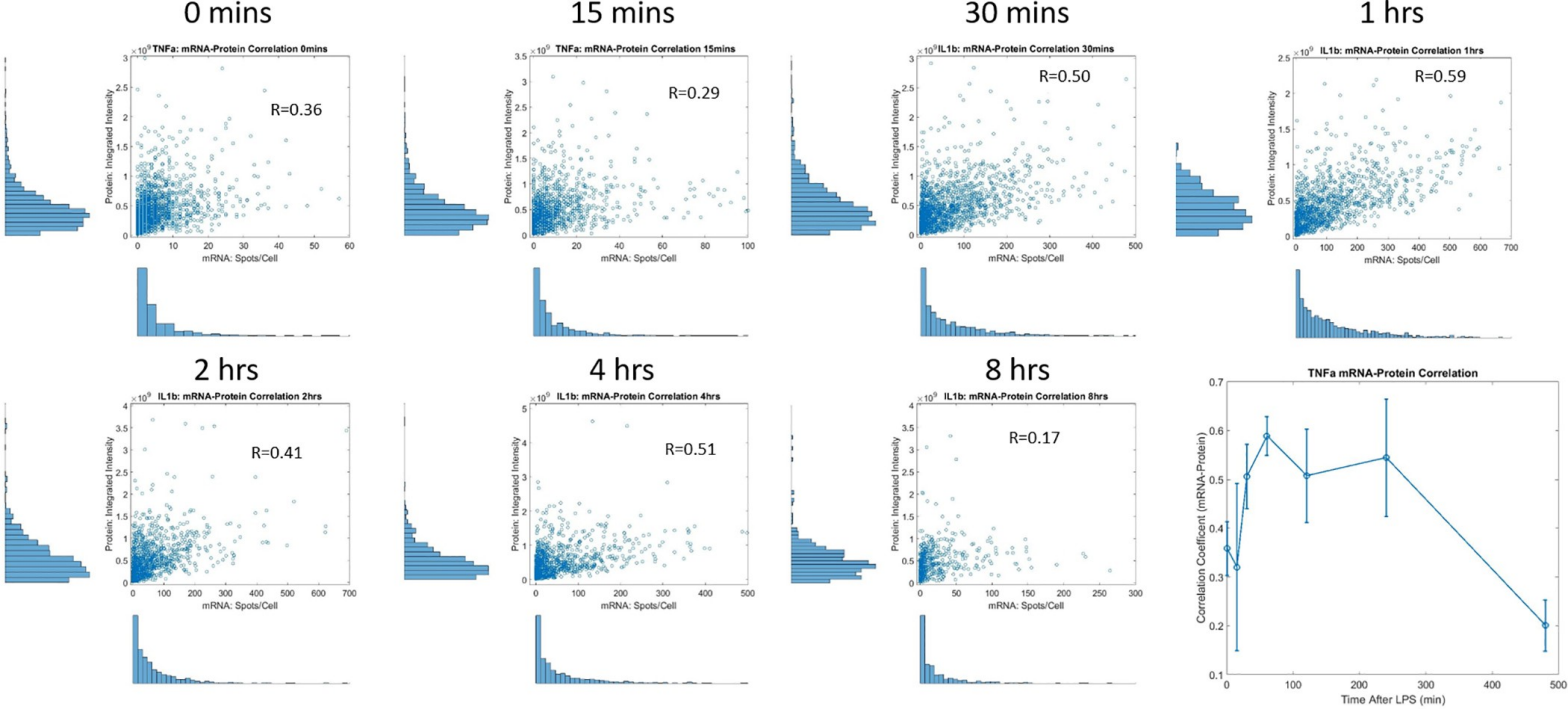
Scatter plots of TNF-α mRNA-Protein data and correlation plot over time.

As shown in Fig 8, we see that correlation between mRNA-protein for IL1β steadily increases over time. In contrast, the correlation between TNF-α mRNA and protein peaks at ~60 minutes then declines at the later time points.(Fig 9) This suggests that TNF-α has a more rapid turnover (with a positive correlation emerging early before the mRNA has yet to decay). We posit that the correlation of IL1β mRNA and protein, which emerges later, could be due to the fact that it takes longer for the protein to turn on and that the IL1β mRNA may be longer lived (and/or that IL1β transcription continues longer post-LPS stimulation).

## Discussion

We have developed an automated platform for the quantification of gene expression to examine the response of IL1β and TNF-α to LPS over time at the single-cell level. Our results with THP-1 immune cells demonstrate a rapid immune response to LPS stimulation and a complex relation between the genes. As expected, the first sign of gene expression for both IL1β and TNF-α is visible at the intron bursting sites located in the cell nuclei, followed by subsequent expression as mature mRNA predominantly in the cytoplasm. Additionally, the correlations of mRNA-mRNA content at the single-cell level demonstrate that IL1β and TNF-α are uncorrelated without LPS stimulation, but become more correlated as gene expression peaks (~1hr). This switch in correlation over time suggests that the external stimulus (LPS) independently activates *both* IL1β and TNF-α upstream, with these genes initially having little interplay between each other.

IL1β and TNF-α regulate inflammatory responses in a variety of diseases and infections. Both genes are examples of immediate-early rapidly-induced genes whose expression is thought to be controlled by a paused RNA polymerase II poised near the transcription start site waiting for activation by specific stimuli.[30] However, IL1β and TNF-α have been reported to exhibit different mechanisms for induction and tolerance in host immune response. [30] In response to stimulus, TNF-α is thought to be only transcribed once, while IL1 β is transcribed multiple times.[25] Upon subsequent LPS exposure, tolerized TNF-α remained in an unresponsive state, whereas IL1β resumed transcription.[30] These observations are consistent with our finding that TNF-α mRNA content peaks shortly after LPS exposure (1 hr), while IL1β has a similar initial reaction, but continues to be transcribed and peaks significantly later in time (4 hrs). TNF-α has been reported to display rapid induction and complete transcription shut down within a few hours of LPS treatment.[30] In contrast IL1β was also rapidly induced, but not completely switched off, with continued expression seen for many hours post-stimulation. Adamik et al further suggested that sustained expression of IL1β resulted from continuous polymerase engagement and not from increased mRNA stabilization. This interpretation agrees with our observations (Fig 3), which could be explained by the fact that IL1β remains constitutively active after the first ~1hr.

The single-cell distributions of both mRNA and intron bursting sites appeared to exhibit ‘bursting’ behavior, characterized by distributions that are similar to an exponential decay, with long tails of relatively rare highly response cells. While the means and decay rates of these distributions change over time (Figs 2 and 6), even at high expression, the basic bursting shape persists. This could suggest that the genes are never fully ‘ON,’ but instead they are still bursting with a different kinetic rate (more time in ON state compared to no LPS).

The absolute value of mRNA expression change is similar to that quantified with qPCR (S11 Fig), while the shapes of the distributions reflect bursting behavior, which suggests that, even when turned ‘ON,’ the genes are produced in a ‘bursting mode’ as opposed to constitutive expression. While bursting sites around the transcription site were readily visible, lone introns for either TNF-α or IL1β were rare. This suggests that the splicing of IL1β and TNF-α occurred rapidly around the same spatial location as the transcription site. Additionally, it should be noted that the spatial location of the intron busting sites for both genes, particularly IL1β, were generally near the edge of the nucleus. This location could be strategically positioned for the rapid transmission of the mRNA into the cytoplasm.

Current drugs are being designed to target IL1β and TNF-α at the protein level. While this is a reasonable and sensible effort, recent work has demonstrated the high potential of targeting RNA.[31] Compounds that have a potential to block transcription at the intron/mRNA level could prove to be significant breakthroughs as therapeutics, and could be successfully tested in an integrated platform such as ours.

We note the single-cell methodologies developed and exploited herein can be used for ultrasensitive measurement of rare genes of interest. While it can be easy to lose the signal from such “outlier” cells/genes in a bulk measurement that requires amplification techniques, our platform allows for direct quantification of such rare events.

## Conclusion

An automated platform for acquisition and quantification of gene expression at the single-cell level has been developed and demonstrated. The kinetic expression of IL1β and TNF-α content is measured with the quantification of intron bursting sites, single-molecule mRNA counting, and protein content. When exposed to LPS, we see a rapid ON time of intron bursting sites (~15 min) followed by an increase in mRNA expression (max at 1hr TNF-α, 4hr IL1β). While initially uncorrelated, the mRNA expression of IL1β and TNF-α at the single-cell level become correlated as gene expression peaks, a result that suggests that the regulation of each gene is independent of each other. This automated platform has the potential to be applied to a variety of single-cell assays where ultrasensitive and quantitative measurement of genes is critical such as cancer cell development, drug response, and persistent bacterial infection.

## Supporting information

S1 Table Segmentation comparison: Manual vs automated. The comparison of manual and automated segmentation processes shows that each yield similar IL1β- TNFα mRNA-mRNA correlation plots, similar distribution shapes, and similar values of mRNA content.
(DOCX)

S1 File RNA FISH probes. Stellaris custom RNA FISH probe sequences and their fluorescent conjugates are detailed in this file. All probes are specific to mRNA unless otherwise stated.
(DOCX)

S2 File Excel file containing raw data to recreate Fig 2, Figs 3 and 4, Figs 6–9. (XLSX)

S1 Fig Selection of smFISH threshold. Following Laplacian-of-Gaussian filtering on the raw image data, the number of spots in the image across over a wide range of thresholds are calculated and plotted (Above). Additionally, the derivate of this plot (the change in spots/Threshold) is calculated to further evaluate the threshold value where the number of spots/threshold begins to become more constant. Beyond the above plots, the threshold values are examined by eye (overlap selected spots with raw image data) to confirm that a suitable threshold value has been selected. Finally, post-processing the fits of the spots can be utilized to gate out spots that have poor fit quality (such as spots with too low/high amplitude/fluorescent intensity, or too narrow/wide a width).
(TIF)

S2 Fig Segmentation comparison: Manual vs automated mRNA scatter plots. Scatter plot of single-cell mRNA correlation between IL1β and TNF-α with automated (blue) and manual (red) segmentation.
(TIF)

S3 Fig Segmentation comparison: Manual vs automated mRNA histograms. Histograms of single-cell mRNA expression for IL1β and TNF-α with manual and automated segmentation are consistent, with minimal deviations resulting from the segmentation process.
(TIF)

S4 Fig Demonstration of cell boundaries determined by automated cell segmentation. Example of automated segmentation, IL1β, TNF-α, and DAPI after 1 hour of LPS Stimulation.
(TIF)

S5 Fig Fits to the single cell mRNA distributions (no stimulation). Fits of single-cell distributions shown in Fig2 without LPS. These single-cell distributions are poorly characterized by a Poisson distribution and reasonably characterized by both Log-normal and Gamma distributions.
(TIF)

S6 Fig Fits to the single cell mRNA distributions (after LPS stimulation). Fits of single-cell distributions shown in Fig 2 with LPS. These single-cell distributions are reasonably characterized by both Log-normal and Gamma distributions. The Poisson distribution poorly fits the single-cell mRNA data.
(TIF)

S7 Fig Reproducibility of mRNA content vs time. Three additional biological replicates of IL1β and TNFα content. In each biological replicate (A, B, and C), cells are seeded at various times across a few months of experiments. We see consistency across the biological replicates, both in terms of absolute mRNA counts and the time of peak expression.
(TIF)

S8 Fig Intron bursting site size. Comparison of intron bursting sites (A,C) to single-mRNA copies (B,D). Gaussian fit of the intensity of bursting sites are ~20 times brighter (amplitude) and ~2–4 times wider (sigma) than single mRNA copies. Integrated intensity under curve is a factor of ~100 larger for bursting sites than single-mRNA copies.
(TIF)

S9 Fig mRNA-Area correlation. The correlation between cell area and mRNA counts for IL1β and TNFα. While there is come correlation between area and mRNA content, it is not as strong as peak mRNA-mRNA correlations (R = 0.80). Additionally, we see that the mRNA-mRNA correlations change over time.
(TIF)

S10 Fig Raw intron image: For comparison to filtered intron image. Raw unfiltered image for intron-staining (left) and the correlation of intron bursting sites over time for IL1β and TNF-α.
(TIF)

S11 Fig Comparison between smFISH and qPCR. While there are some discrepancies in the absolute value, we see general agreement between the bulk qPCR and mRNA data from single-cell smFISH measurements.
(TIF)
