# Supplementary material for: Single-cell correlations of mRNA and protein content in a human monocytic cell line after LPS stimulation
Source: PLoS One. 2019 Apr 19;14(4):e0215602. doi: 10.1371/journal.pone.0215602 (PMC6474627; doi:10.1371/journal.pone.0215602)
Supplement: S1 Table — The comparison of manual and automated segmentation processes shows that each yield similar IL1β- TNFα mRNA-mRNA correlation plots, similar distribution shapes, and similar values of mRNA content. (DOCX) [file pone.0215602.s001.docx]

**S5 Table**

|  | Manual Segmentation | Automated Segmentation |
| --- | --- | --- |
| Total Number Cells | 289 | 272 |
| IL1β-TNFα mRNA Correlation | 0.7364 | 0.7639 |
| Mean IL1β mRNA Counts | 371.84 | 395.09 |
| Mean TNFα mRNA Counts | 274.53 | 292.85 |
| Stdev IL1β mRNA Counts | 301.77 | 316.53 |
| Stdev TNFα mRNA Counts | 199.61 | 203.12 |
| Cell Area –mRNA Correlation IL1β | 0.5805 | 0.642 |
| Cell Area –mRNA Correlation TNFα | 0.7286 | 0.692 |
