# Supplementary material for: Single-cell correlations of mRNA and protein content in a human monocytic cell line after LPS stimulation
Source: PLoS One. 2019 Apr 19;14(4):e0215602. doi: 10.1371/journal.pone.0215602 (PMC6474627; doi:10.1371/journal.pone.0215602)
Supplement: S1 File — Stellaris custom RNA FISH probe sequences and their fluorescent conjugates are detailed in this file. All probes are specific to mRNA unless otherwise stated. (DOCX) [file pone.0215602.s002.docx]

**S6 File**

Stellaris custom RNA FISH probe sequences and their fluorescent conjugates are detailed below. All probes are specific to mRNA unless otherwise stated.

Oligo Name: **IL1-beta**
Three Prime: CAL Fluor Red **610**
Size/Scale: 5 nmol Total
Sequence: ttctctgttgaatacctga ctcgctgtttttatggctt ttggtatctgccagtttct tgccttgtgcctcgaagag ggctgaagagaatcccaga tgcttcagacacttgagca ctcaggtacttctgccatg aagccatcatttcactggc aagtcatcctcattgccac tagggccatcagcttcaaa cctggaaggagcacttcat catccagagggcagaggtc cggagattcgtagctggat tgaagcccttgctgtagtg tggccacaacaactgacgc agcatcttcctcagcttgt aaggtctgtgggcagggaa gttatcccatgtgtcgaag ggtgcatcgtgcacataag gtgcagttcagtgatcgta caccaagcttttttgctgt agattcttttccttgaggc ttcaacacgcaggacaggt ctgtagagtgggcttatca tttgggatctacactctcc ctgggcagactcaaattcc tgagaggtgctgatgtacc caggaagacgggcatgttt aagtcagttatatcctggc ggtacagctctctttagga cattcagcacaggactctc cagccctagggattgagtc aaacctttctgttcccttt ggaaagtccaggctatagc ttgggcattggtgtagaca tcttagcactaccctaagg tggctgatggacaggagat ccctgaaaggagagagctg cctggctcaacaaaagggc caggcgggctttaagtgag agaaccaaatgtggccgtg ggagcgaatgacagagggt gcggttgctcatcagaatg agacactgctacttcttgc gcacaccagtccaaattga

Oligo Name: **TNF-alpha**
Three Prime: **Quasar 670**
Size/Scale: 5 nmol Total
Sequence: ggtctgtagttgcttctctc cgtctgagggttgttttcag gggtcagtatgtgagaggaa atcatgctttcagtgctcat caaagtgcagcaggcagaag gagggctgattagagagagg cggggttcgagaagatgatc cttgagggtttgctacaaca accagctggttatctctcag ctgggagtagatgaggtaca ctgatggtgtgggtgaggag tctggtaggagacggcgatg ctcttgatggcagagaggag gatagatgggctcataccag attgatctcagcgctgagtc caaagtcgagatagtcgggc aaagtagacctgcccagact ctcctcacagggcaatgatc ttgggaaggttggatgttcg ggtaataaagggattggggc cagaagaggttgagggtgtc aagttctaagcttgggttcc tcgaagtggtggtcttgttg cacacattcctgaatcccag ttgaattcttagtggttgcc agggatcaaagctgtaggcc tggtctccagattccagatg cattctggccagaaccaaag taggtgaggtcttctcaagt aaggtccacttgtgtcaatt acatctggagagaggaaggc cgtgtctcaaggaagtctgg caaggcagctcctacattgg agctccgttttcacggaaaa ctacatgggaacagcctatt caaaagaaggcacagaggcc ttggtcaccaaatcagcatt agaggctcagcaatgagtga gggcgattacagacacaact ctttatttctcgccactgaa

Oligo Name: **IL1-beta introns**
Three Prime: **Quasar** **570**
Size/Scale: 5 nmol Total
Sequence: gaaagaggagacctgctcat gcacagataacactctactc aggggaaaaatctggtctcc tttgccactgaaagaggcaa aatcagatttacttggcacc tggagggacttgtaatggga acaacacatctggatagcct actttgcaaagggattcctt ccagcagagtcagctaaaat tgttggatcttgaggcctaa aacagtccagaaggcagatt agtttcacagtgatatgcgc aaagctgcctgaaacacctg agaaactgcaaacagcctgc cctgtgtacaagacttgact gggacatgtctcaattatgt tgggagttaaacccatagtc atcaaagtggcccagaactc tgtctcactggaagaggtta atatgatgaccaagtggcca acgttagtgagtgactgtgg aacattatcactactcctcc ttggttgtcttcctactatg taggctgaatccatctctta catctcttggtttcagagtt ggggtagacatatgttagtt caccccaggtagcaaaaaac atcatctatgtcttgttgtt gtctcaattcatctttttgt caaatgcccttagctacaac ggctctttgtaaggatgagt acagaagggacagcaggaat taggaaggctactgagcatc tgcacaactgacctagctag cagagaaaagtggctgcctc ggactggattatgaggctag caacaagttatgccagtctt aagaggcaatgggggcaaag gagcaggagagaatttcctg agagacctgtaagtgcacaa gttgccttaaggcatgataa tccgtattaaggcttcactg ttaccactttcctcttattc ctatgctcactcagcaagta ctttttcatgcagatgacca ccacctacaccatacatgta atcccgagcttctaaagatc accatgctttgacatcagag

Oligo Name: **TNF-alpha**

Three Prime: **Quasar** **570**
Size/Scale: 5 nmol Total
Sequence: ggtctgtagttgcttctctc cgtctgagggttgttttcag gggtcagtatgtgagaggaa atcatgctttcagtgctcat caaagtgcagcaggcagaag gagggctgattagagagagg cggggttcgagaagatgatc cttgagggtttgctacaaca accagctggttatctctcag ctgggagtagatgaggtaca ctgatggtgtgggtgaggag tctggtaggagacggcgatg ctcttgatggcagagaggag gatagatgggctcataccag attgatctcagcgctgagtc caaagtcgagatagtcgggc aaagtagacctgcccagact ctcctcacagggcaatgatc ttgggaaggttggatgttcg ggtaataaagggattggggc cagaagaggttgagggtgtc aagttctaagcttgggttcc tcgaagtggtggtcttgttg cacacattcctgaatcccag ttgaattcttagtggttgcc agggatcaaagctgtaggcc tggtctccagattccagatg cattctggccagaaccaaag taggtgaggtcttctcaagt aaggtccacttgtgtcaatt acatctggagagaggaaggc cgtgtctcaaggaagtctgg caaggcagctcctacattgg agctccgttttcacggaaaa ctacatgggaacagcctatt caaaagaaggcacagaggcc ttggtcaccaaatcagcatt agaggctcagcaatgagtga gggcgattacagacacaact ctttatttctcgccactgaa

Oligo Name: **TNF-alpha introns**

Three Prime: CAL Fluor Red **610**
Size/Scale: 5 nmol Total
Sequence: tgaaggctggccaggcac cccttgggtgggagagtg ctgcttgttcattcattc catctgtctccatatctt atctctcttctcacaccc tatcacttgtttcttccc ctttctgtctcaccatct tagctgtcatatttcccg ttatctcccccatctctc caccctatcttcttctct agtgtcttctgtgtgcca attcaacagctctttccc ctgtgtattcaccttcca ggttttctctctccattc ctcttagccctgaggtgt aggaggaacagctggctg acatcgagggagtcaccc ggggagaaggagaatggt ggtttggagacacttacc ccaaacccaaacccagaa cataccggtactaaccct taaatttcccccactgct ctcccccaagaccaaaac aaatacccccctactttc gagacccttaaacttcct ctcagagctcttacctga cctccaagttccaagaca ggcttcaatccccaaatc agttctgcctaccatcag tccttctcacattgtctc cttcccttgagctcagcg actaaggcctgtgctgtt gccatgacgttctgagta tgtcatcccacatcccac cggttcctgtcctctctg ttcactctccacatcctg agtcagtgtggccatgtc
