# Supplementary figures and images for: Single-cell correlations of mRNA and protein content in a human monocytic cell line after LPS stimulation
Source: PLoS One. 2019 Apr 19;14(4):e0215602. doi: 10.1371/journal.pone.0215602 (PMC6474627; doi:10.1371/journal.pone.0215602)

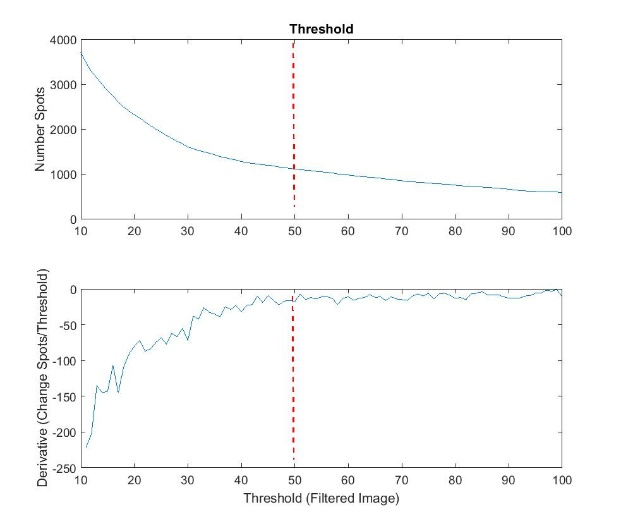

Supplement: S1 Fig — Following Laplacian-of-Gaussian filtering on the raw image data, the number of spots in the image across over a wide range of thresholds are calculated and plotted (Above). Additionally, the derivate of this plot (the change in spots/Threshold) is calculated to further evaluate the threshold value where the number of spots/threshold begins to become more constant. Beyond the above plots, the threshold values are examined by eye (overlap selected spots with raw image data) to confirm that a suitable threshold value has been selected. Finally, post-processing the fits of the spots can be utilized to gate out spots that have poor fit quality (such as spots with too low/high amplitude/fluorescent intensity, or too narrow/wide a width). (TIF) [file pone.0215602.s004.tif]

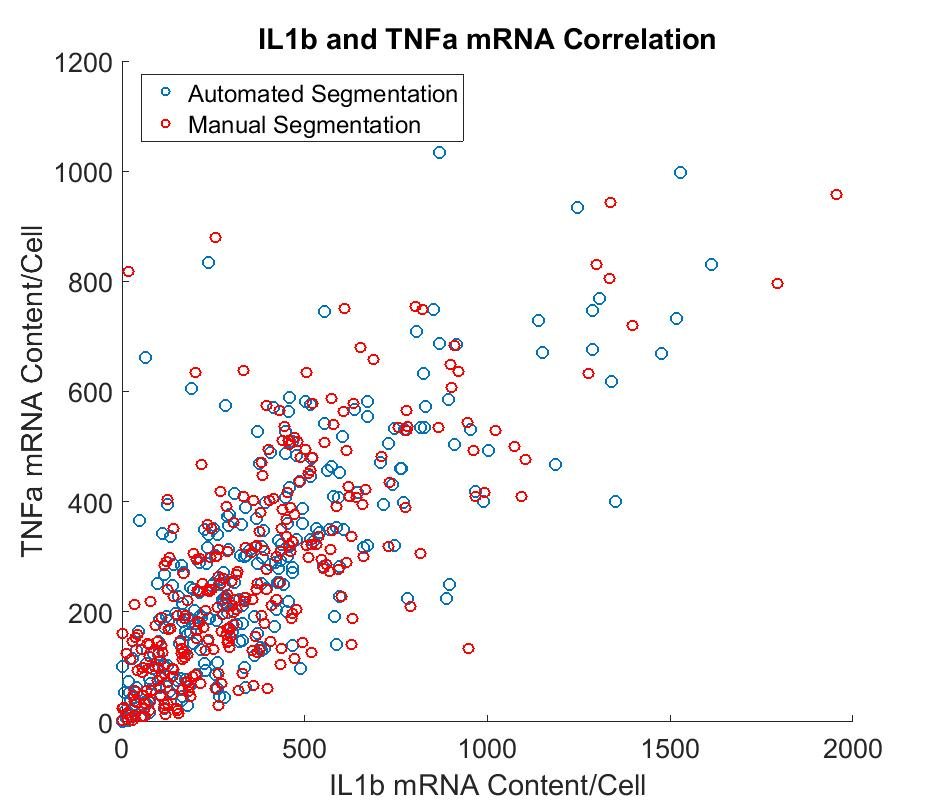

Supplement: S2 Fig — Scatter plot of single-cell mRNA correlation between IL1β and TNF-α with automated (blue) and manual (red) segmentation. (TIF) [file pone.0215602.s005.tif]

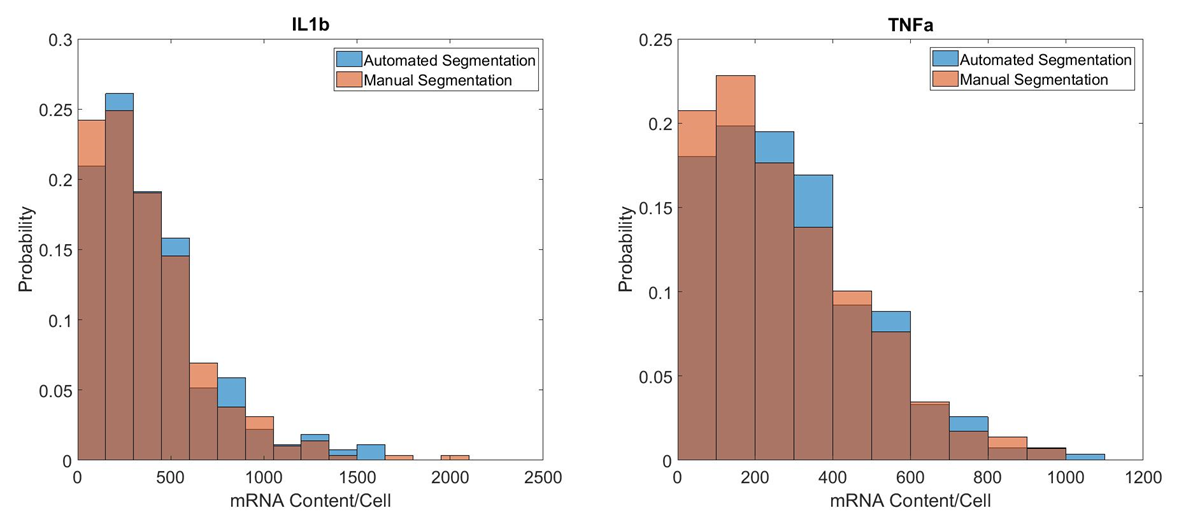

Supplement: S3 Fig — Histograms of single-cell mRNA expression for IL1β and TNF-α with manual and automated segmentation are consistent, with minimal deviations resulting from the segmentation process. (TIF) [file pone.0215602.s006.tif]

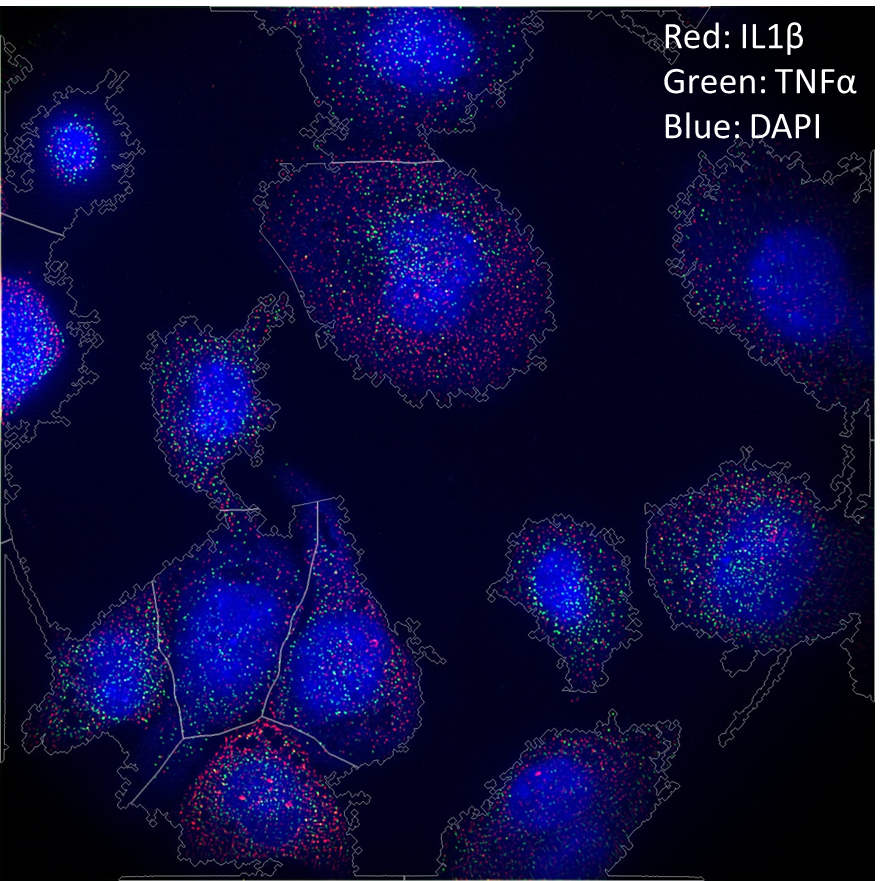

Supplement: S4 Fig — Example of automated segmentation, IL1β, TNF-α, and DAPI after 1 hour of LPS Stimulation. (TIF) [file pone.0215602.s007.tif]

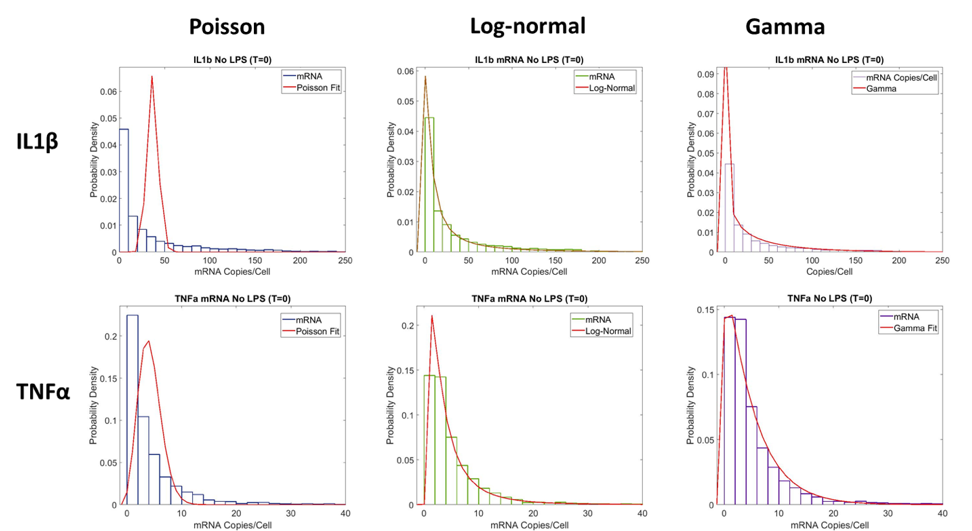

Supplement: S5 Fig — Fits of single-cell distributions shown in Fig2 without LPS. These single-cell distributions are poorly characterized by a Poisson distribution and reasonably characterized by both Log-normal and Gamma distributions. (TIF) [file pone.0215602.s008.tif]

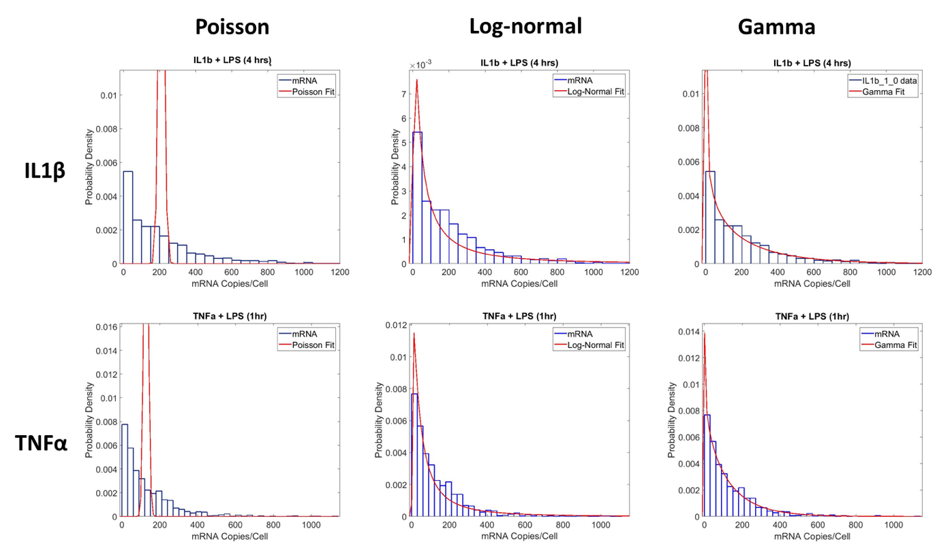

Supplement: S6 Fig — Fits of single-cell distributions shown in Fig 2 with LPS. These single-cell distributions are reasonably characterized by both Log-normal and Gamma distributions. The Poisson distribution poorly fits the single-cell mRNA data. (TIF) [file pone.0215602.s009.tif]

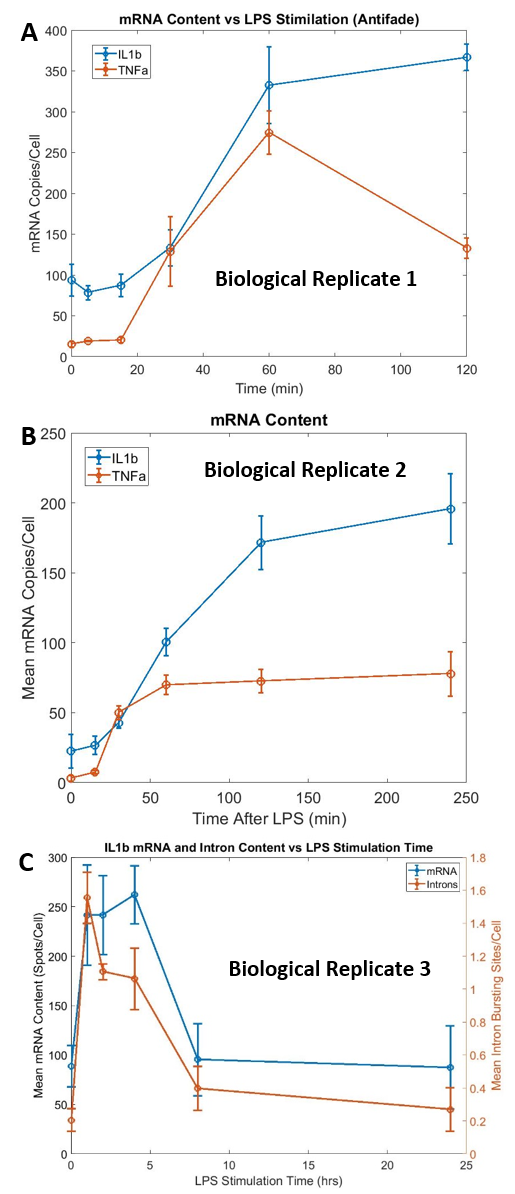

Supplement: S7 Fig — Three additional biological replicates of IL1β and TNFα content. In each biological replicate (A, B, and C), cells are seeded at various times across a few months of experiments. We see consistency across the biological replicates, both in terms of absolute mRNA counts and the time of peak expression. (TIF) [file pone.0215602.s010.tif]

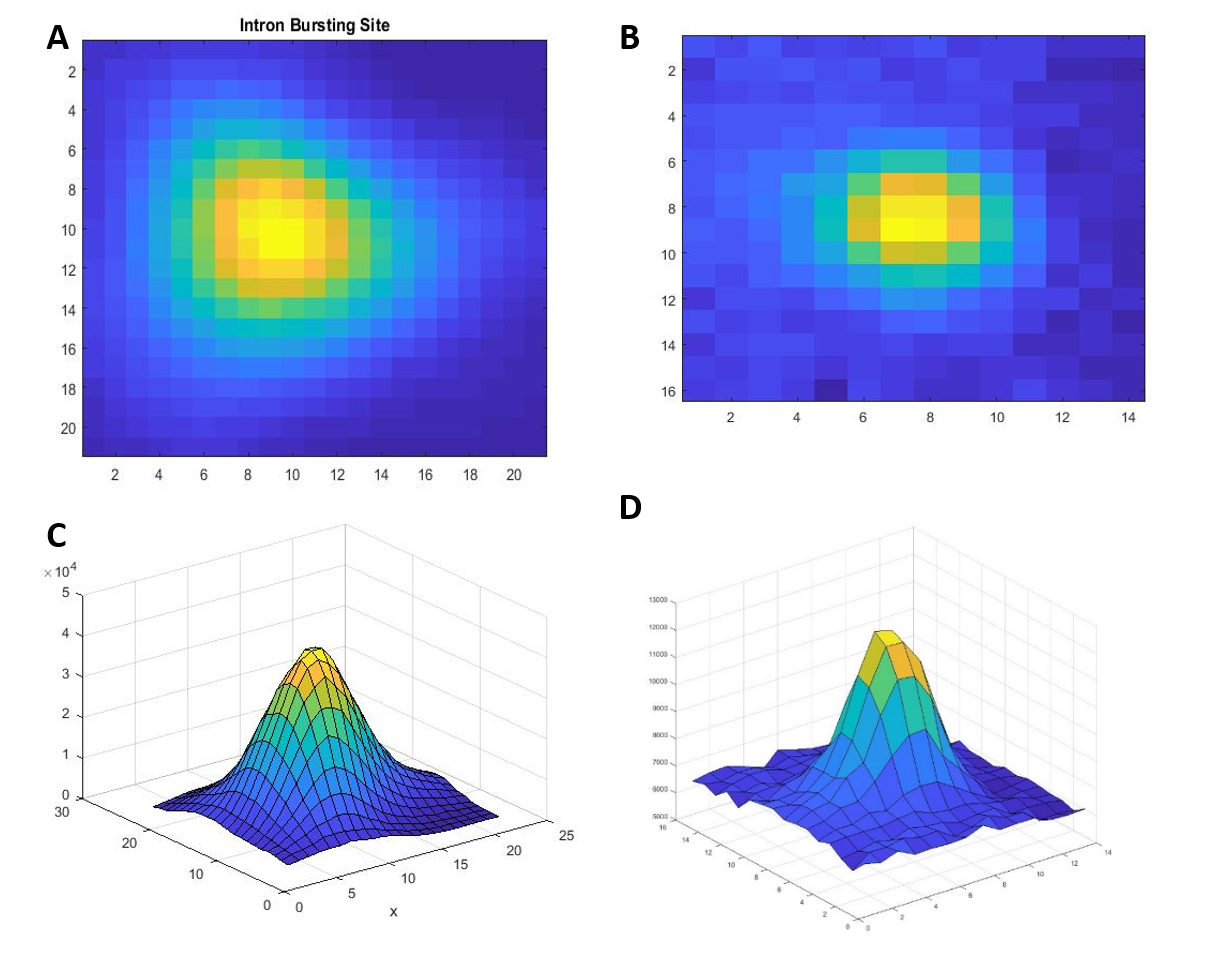

Supplement: S8 Fig — Comparison of intron bursting sites (A,C) to single-mRNA copies (B,D). Gaussian fit of the intensity of bursting sites are ~20 times brighter (amplitude) and ~2–4 times wider (sigma) than single mRNA copies. Integrated intensity under curve is a factor of ~100 larger for bursting sites than single-mRNA copies. (TIF) [file pone.0215602.s011.tif]

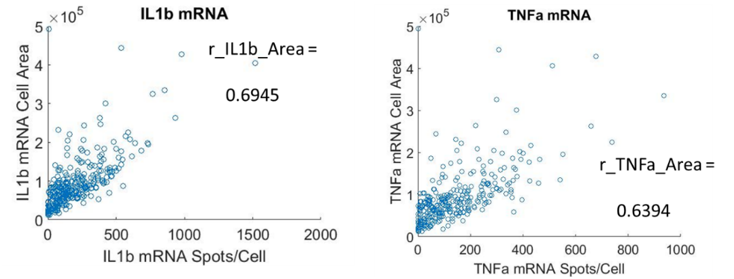

Supplement: S9 Fig — The correlation between cell area and mRNA counts for IL1β and TNFα. While there is come correlation between area and mRNA content, it is not as strong as peak mRNA-mRNA correlations (R = 0.80). Additionally, we see that the mRNA-mRNA correlations change over time. (TIF) [file pone.0215602.s012.tif]

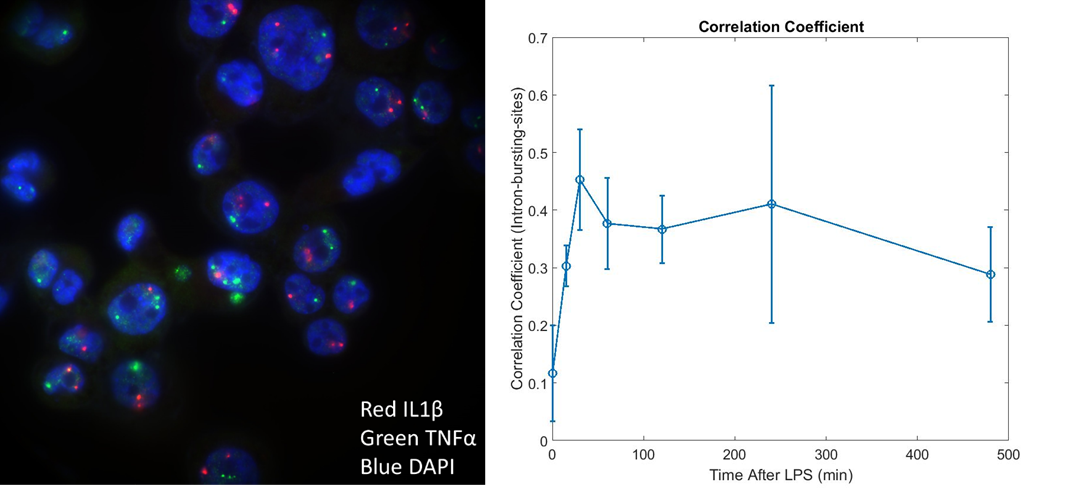

Supplement: S10 Fig — Raw unfiltered image for intron-staining (left) and the correlation of intron bursting sites over time for IL1β and TNF-α. (TIF) [file pone.0215602.s013.tif]

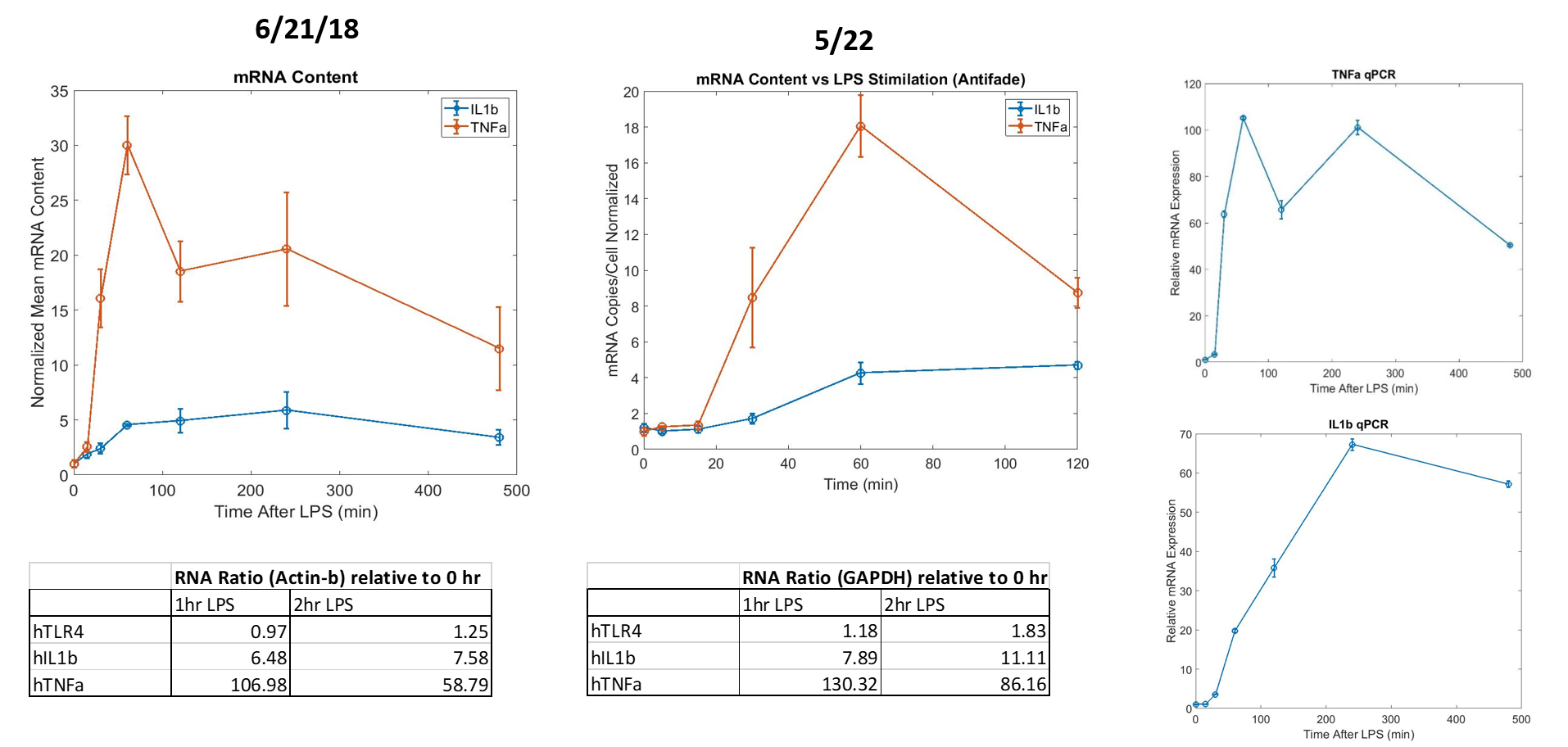

Supplement: S11 Fig — While there are some discrepancies in the absolute value, we see general agreement between the bulk qPCR and mRNA data from single-cell smFISH measurements. (TIF) [file pone.0215602.s014.tif]
